# Solvatochromism as a Novel Tool to Enumerate the Optical and Luminescence Properties of Plastic Waste Derived Carbon Nanodots and Their Activated Counterparts

**DOI:** 10.3390/nano13081398

**Published:** 2023-04-18

**Authors:** Savita Chaudhary, Manisha Kumari, Pooja Chauhan, Ganga Ram Chaudhary, Ahmad Umar, Sheikh Akbar, Sotirios Baskoutas

**Affiliations:** 1 Department of Chemistry and Centre of Advanced Studies in Chemistry, Panjab University, Chandigarh 160014, India; 2 Centre for Scientific and Engineering Research, Najran University, Najran 11001, Saudi Arabia; 3 Department of Chemistry, Faculty of Science and Arts, and Promising Centre for Sensors and Electronic Devices (PCSED), Najran University, Najran 11001, Saudi Arabia; 4 Department of Materials Science and Engineering, The Ohio State University, Columbus, OH 43210, USA; 5 Department of Materials Science, University of Patras, 26500 Patras, Greece; bask@upatras.gr

**Keywords:** carbon dots, activated carbon, transition state, excitation emission, stoke shift

## Abstract

Herein, we have developed a one-pot methodology to synthesise three types of C-dots and their activated counterparts from three different types of waste plastic precursors such as poly-bags, cups and bottles. The optical studies have shown the significant change in the absorption edge in case of C-dots in comparison to their activated counterparts. The respective variation in the sizes is correlated with the change in electronic band gap values of formed particles. The changes in the luminescence behaviour are also correlated with transitions from the edge of the core of formed particles. The obtained variations in the Stokes shift values of C-dots, and their ACs were used to explore the types of surface states and their related transitions in particles. The mode of interaction between C-dots and their ACs was also determined using solvent-dependent fluorescence spectroscopy. This detailed investigation could provide significant insight on the emission behaviour and the potential usage of formed particles as an effective fluorescent probe in sensing applications.

## 1. Introduction

The immense popularity of carbon-based nanomaterials and activated nanofibres has mainly arisen due to their unique optical and mechanical properties [1,2]. The high thermal photostability of carbon-based nanomaterials has further enhanced their potentials in optoelectronic gadgets and as biological markers [3,4]. The comparatively high surface area with superior electrical conductivity, flexible nature and high porosity of activated carbon fibers (ACF) has made them a suitable contender for preparing electrodes in supercapacitors [5]. The tunable optical properties of carbon-based nanomaterials have further attracted their significance in nonlinear optical devices. Additionally, the high surface area of carbon-based nanomaterials and activated nanofibres have been explored comprehensively for their wide range of applications in adsorptive removal of industrial toxins from wastewater resources [6,7]. Activated carbon nanofibres and nanodots have also provided a good porosity and surface area for interfacial interactions with solvent media by modulating the optical and luminescence properties via solvatochromism. The excitation-dependent fluorescence emission with high quantum yield values has enhanced their environmental sensitivity and selectivity aptitude towards harmful toxins [8,9]. Therefore, the characteristic importance of carbon-based nanomaterials and activated nanofibers is mainly due to their strong and tunable photoluminescence. The widespread literature signifies that researchers have paid considerable effort to explore the fundamental emission mechanisms associated with C-dots. This behavioral aspect was scrutinized by investigating the effect of surface groups, pH and kinetics of charge transference among the surrounding ions [10,11]. However, the exact emission mechanisms in C-dots are still a topic of debate. A few authors explained the phenomena via the electronic transitions in the core structure of C-dots; however, some have associated it with the surface-defect states in C-dots [12,13,14]. The involvement of surface states in emission mechanisms has further widened the viewpoint of researchers towards exploring their optical and luminescence properties in different solvent media.

For instance, He et al. described the systematic studies on the emission properties of C-dots derived from 2,7-naphthalenediol and dimethylformamide as precursor sources. The results of both the steady-state and transient-state emissions have further been compared w.r.t. variations in the environment and the stability of the particles under different reaction conditions [15]. Mohammad-Jafarieh et al. used the application of persimmon peels for the synthesis of C-dots and investigated the effect of different ranges of solvents on the emission and optical properties. The changes in the quantum yield values of obtained particles in different solvent systems have also been compared to obtain insight into the photo-physical behavior of C-dots [16]. The fabrication of narrow bandwidth emissive C-dots has been carried out by Yoshinaga et al. [17]. The formed particles displayed the shifting of emission peaks from 463 to 511 nm as a function of solvent polarities. The emission color also changed from blue to green via taking advantage of solvatochromic phenomena and enhancing the scope of formed particles in wide color gamuts. 

These solvent-dependent optical and luminescence studies could enhance awareness about the photophysical properties of carbon quantum dots (CQDs) and be useful to fulfill the existing knowledge gaps where only a few papers have discussed the influence of a diverse range of solvents on the emission properties and discussed their solvatochromic effect. However, no report has compared the optical and luminescence properties of C-dots and their activated counterparts in terms of solvatochromism.

In this outlook, the current work tackles these issues and investigates the solvent interactions with three different types of C-dots derived from single-use plastic waste including cups, bottles and polybags for elucidating the nature of their optical and emission properties. In order to authenticate the proposed idea, the activated C-dots (ACs) prepared from single-used plastic cups, bottles and poly-bags have also been studied as the initial target models in solvatochromic experiments. We scrutinised and compared the optical and emission spectral data for the above-mentioned carbon-based nanodots and their activated counterparts in solvents with a diverse range of polarities. The simplicity of the used procedure with high sensitivity has opened a new pathway to its application in a diverse range of analytical devices in bio-imaging, diagnostics and environmental remediation applications.

## 2. Experimental Details

### 2.1. Reagents

Methanol (CH_3_OH), hydrogen chloride (HCl), sulphuric acid (H_2_SO_4_), ethanol (C_2_H_5_OH) and sodium hydroxide (NaOH) were procured from Fisher Scientific with 99% purity. Single-use plastic waste such as cups, bottles and poly-bags were acquired from the Department of Chemistry, Panjab University, Chandigarh, India. Chloroform, ethanol, acetonitrile, dimethylformamide, dichloromethane, acetic acid, methanol, acetone, cyclohexane and toluene were taken from Fisher Scientific with 99% purity for solvatochromic studies. The deionized water was utilized for the preparation of all the aqueous solutions. The analytical grade reagents and chemicals were utilized without any further treatment. 

### 2.2. Instrumentation

Fourier transform infrared (FTIR) spectroscopy (Perkin Elmer (RX1)) was utilised to investigate the presence of different functional groups on the surface of developed C-dots and ACs from different plastic sources in the range 500–3500 cm^−1^. The absorption performance of formed particles was carried out by using LABINDIA UV-visible spectroscopy in the range 200–800 nm with a fixed path length 1.0 cm. A Perkin Elmer LS 55 PL spectrometer was utilized to investigate the fluorescence emission profile of developed C-dots and ACs in the wavelength range 200–800 nm. An AICIL muffle furnace was utilized to carry out the thermal calcination process of utilized plastic precursors. A Remi ultracentrifugation instrument was utilized to separate the aggregated particles. The surface morphology of synthesized C-dots and ACs was analyzed on a High-Resolution Transmission Electron Microscope (HRTEM, Hitachi H7500 electron microscope) (Tokyo, Japan) at 100 kV. Thermogravimetric Analysis was conducted on a Mettler Toledo (Q20) instrument. Magnetic stirring was performed using an IKA C-MAG HS 7 stirrer instrument. Crystalline size and phases of developed samples were evaluated using an XRD powder diffractometer (Panalytical X’Pert Pro) instrument utilizing scan rate of 2 deg. min^−1^ with Cu-Kα radiation.

### 2.3. Fabrication of C-Dots Using Single-Use Plastic Waste

The current work demonstrated the simple synthesis of C-dots and their counterpart ACs from plastic waste, such as plastic cups, bags and bottles made of polypropylene, polyethylene and polyethylene terephthalate (PET) polymers, respectively. Initially, the three types of highly fluorescent C-dots were fabricated by a simple thermal calcination method employing single-use plastic waste, i.e., cups, bottle and ploybags as precursor sources [18]. For the synthesis, the chosen thoroughly cleaned precursor sources (i.e., cups, bottle and polybags) were placed in a ceramic crucible and thermally calcined at 450 °C for 120 min. The as obtained dark black powder was further crushed mechanically to form fine powder of carbon. Subsequently, 0.5 g of powder dissolved into 100 mL of distilled water under stirring conditions to form a carbonized solution of C-dots. Thereafter, the as-formed colloidal dark black suspension of three different types of C-dots (i.e., B@C-dots, C@C-dots and P@C-dots from waste bottle, cups and poly-bags, respectively) were allowed to ultracentrifuge at 16,000 rpm to obtain solid extracts and used further for optical and structural studies.

### 2.4. Fabrication of ACs Using Single-Use Plastic Waste

The solid extract from three different types of precursor sources was then allowed for chemical activation by using 8 M KOH solution for 1 day. Afterward, the obtained solution from three different types of precursor sources was thoroughly cleaned with water and oven-dried. The as-obtained samples were calcinated at 500 °C for 120 min. Subsequently, the cooled-down samples were thoroughly washed with deionized water to maintain the neutral pH of the reaction medium. Afterward, these three different dispersions were allowed to centrifuge at 5000 rpm and termed as B@ACs, C@ACs and P@ACs (i.e., activated carbon from the waste bottle, cups and poly-bags, respectively). 

## 3. Results and Discussion

### 3.1. Structural and Morphological Characterization

**Figure 1:**
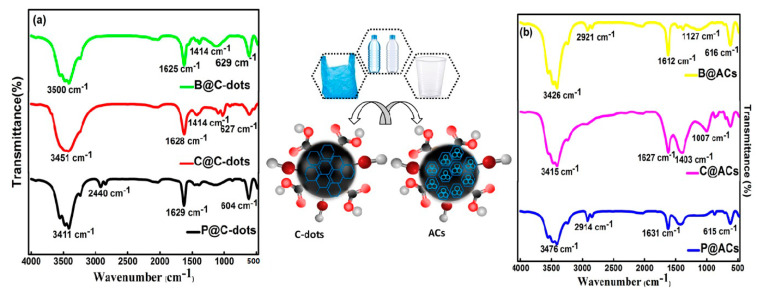
FTIR spectra of (**a**) C-dots and (**b**) ACs derived from single-use plastic bottle, cups and polybags.

The presence of different types of functional groups over the surface of C-dots and their counterpart ACs were assessed using FTIR spectroscopy in region 500–4000 cm^−1^ (Figure 1). The characteristic peaks at 3400–3500 cm^−1^ in both C-dots and ACs have confirmed the presence of −OH stretching mode due to the existence of carboxylic, phenolic and alcohol functional groups present on their exterior surface [19]. However, in the case of C-dots, the other strong transmission bands at 1628 and 1414 cm^−1^ are associated with the −C=C and −C−OH stretching vibrations [20]. However, the small peaks below 700 cm^−1^ in both C-dots and ACs have arisen due to the presence of C=C bending vibrations. In the case of ACs, the intense common absorption peaks around 1600 cm^−1^ are mainly attributed to the existence of C−C, C=O and C=C vibrations from the carboxylic groups, as presented in Table S1 [21].

**Figure 2:**
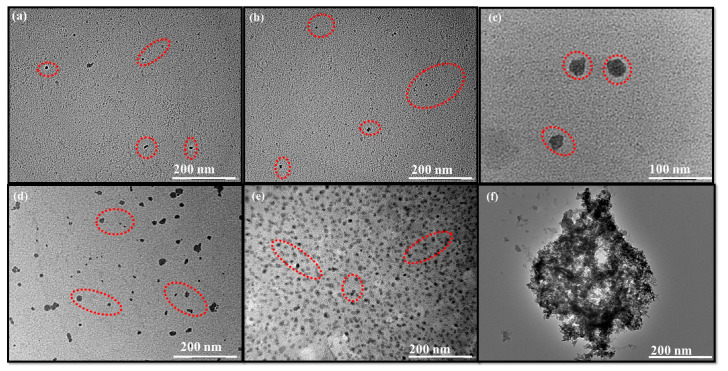
TEM micrographs of (**a**–**c**) P@CQDs, C@CQDs, B@CQDs and (**d**–**f**) P@ACs, C@ACs and B@ACs, respectively.

The X-ray powder diffraction analysis was carried out to explore the crystalline structure of formed C-dots and ACs prepared from single-use plastic (Figure S1). The different peaks at 2θ angle and their corresponding diffraction planes have been represented in Table S2. The crystalline size of developed C-dots was calculated by using the Scherrer equation (D = kλ/βCosϴ) and found to be in the range of 22 nm. The resultant diffraction planes support the crystalline nature and high purity of formed particles. Additionally, the position of peaks in ACs prepared from single-use plastic waste are highly comparable with the standard pattern database (JCPDS No. 721616), which further supports the complete formation of ACs [22]. The slight enhancement in crystalline size in ACs as compared to C-dots was mainly attributed to their higher agglomeration rate. The topographical analysis of prepared C-dots and ACs has been further investigated through TEM analysis (Figure 2). The obtained results suggested the formation of high-density C-dots from all the three precursor sources with average size range between 4–9 nm with excellent dispersibility and homogeneity (Figure 2a–c). However, the case of ACs derived from three different carbon precursors has shown the existence of different size cavities due to the KOH activation process. The obtained size of ACs has also shown the increment to 60–90 nm (Figure 2d–f). 

### 3.2. Optical Properties of Carbon Dots and Their Activated Counterparts 

**Figure 3:**
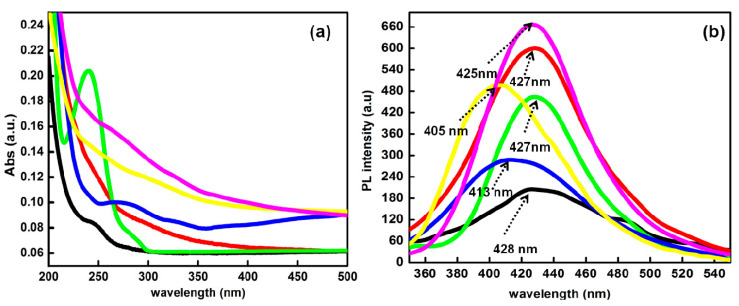
(**a**) UV-visible and (**b**) fluorescence emission profile of C-dots and their counterpart ACs at an excitation wavelength of 310 nm.

The UV–vis. spectra of C-dots and ACs derived from single-use plastic bottles, cups and polybags is shown in Figure 3a. All the prepared particles showed the characteristic optical absorption peak in the UV-region with an end part extended very little towards the visible region. Additionally, the nature of the precursor sources displayed a significant variation on the UV-vis. spectra of all the types of prepared particles. For instance, P@C-dots showed the absorption edge at 244 nm (5.08 eV) and 279 nm (4.44 eV). However, the C@C-dot showed the absorption edges at 246 nm (5.04 eV) and 290 nm (4.27 eV), and the B@C-dot displayed the absorption edge at 240 nm (5.17 eV) and 280 nm (4.43 eV), respectively [23]. However, activated counterparts of C-dots showed the major absorption edges at 274 nm (4.52 eV), 291 nm (4.26 eV) and 255 nm (4.86 eV) for P@ACs, C@ACs and B@ACs, respectively [24]. 

The spectral peaks below 240 nm were mainly explained due to the presence of π-π* transitions for sp^2^ hybridized conjugated carbon rings present inside the sp^3^ matrix. These conjugated rings mainly compose the core domains of the C-dots. The other absorption edges between 260 to 300 nm in all three types of C-dots mainly arise due to the n-π* of –C=O groups originating from the different types of electron-withdrawing groups containing oxygen, including carboxyl or carbonyl types of functional groups over the exterior surface of three different types of C-dots [25]. Additionally, the as-prepared C-dot solution displayed a brownish-yellow color dispersion under visible light and displayed green- blue color fluorescence light in the UV region. The significant change in the absorption edge from C-dots to their activated counterpart is mainly explained due to the change in the size of particles, and corresponding results further affected their electronic band gap values [26]. The broad band is mainly associated with the non-uniform and heterogeneous distribution of particles with different sizes. The red shift arose due to the enhancement in the auxochromic and chromophoric group. 

The photoluminescence behavior of the aqueous dispersion of C-dots and their ACs is depicted in Figure 3b. The respective emission spectra showed distinguishing peaks at 428 nm for P@C-dots, and 427 and 428 nm for C@C-dots and B@C-dots, respectively. However, the peaks for activated counterparts of C-dots showed significant blue shift and the respective peaks were now observed at 413 nm, 405 nm and 425 for P@ACs, B@ACs and C@ACs, respectively, whereas the relative values of Stokes shift of the emission spectra for all the three kinds of C-dots and their respective ACs with reference to the absorption spectra are around 184 nm for P@C-dots, 181 nm for C@C-dots, 187 nm for B@C-dots, 139 nm for P@ACs, 136 nm for B@ACs and 135 nm for C@ACs. The obtained variations in the luminescence are mainly related with the corresponding transitions from the edge of the core of formed particles [27]. In addition, these variations in the Stokes shift values for the prepared particles were further associated with the types of surface states and their related transitions. The changes in the size of the prepared C-dots and their counterpart ACs also affected the changes in PL transitions and related Stokes shift values. Moreover, theses variations also influenced the electronic structure of relaxed and ground state transitions in C-dots and their counterpart ACs. Such behavioural changes have also been used to study the PL transitions in the presence of different solvents [28].

**Figure 4:**
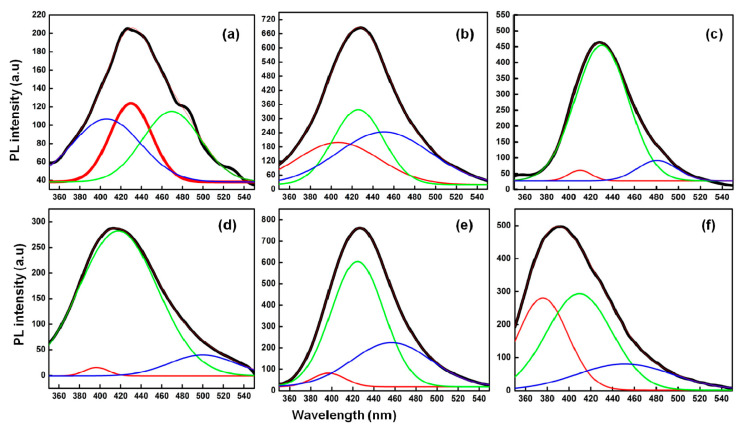
Gaussian analysis of the emission spectra of (**a**) P@C-dots, (**b**) C@C-dots, (**c**) B@C-dots, (**d**) P@ACs, (**e**) C@ACs and (**f**) B@ACs at λ = 310 nm.

The respective Gaussian analyses of the emission peaks for all three types of prepared C-dots and their counterpart ACs were performed to obtain the information about the nature of the defects in the CQDs (Figure 4). The broad peak in all the samples was divided into three peaks. The respective first peak (green line) is associated with the type of defect-bound excitons (1_DBE_). However, the second (red line) and the third peaks (blue line) are associated with the defect-charged excitons (2_DCE_) electron–hole recombination effect (3_EHR_) in formed C-dots and their counterpart ACs [29]. The respective peak positioning, area under the curve and full width at half maxima (FWHM) values for all the samples are given in Table S3. On interpreting the data, it was found that the 1_DBE_, 2_DCE_ and 3_EHR_ show the considerable changes with the change in the nature of formed particles. Additionally, the availability of electron-hole pairs over the surface of prepared particles is different in each case, which further supports the significant changes in the values of positioning, area under the curve and full width at half maxima values for prepared particles. 

**Figure 5:**
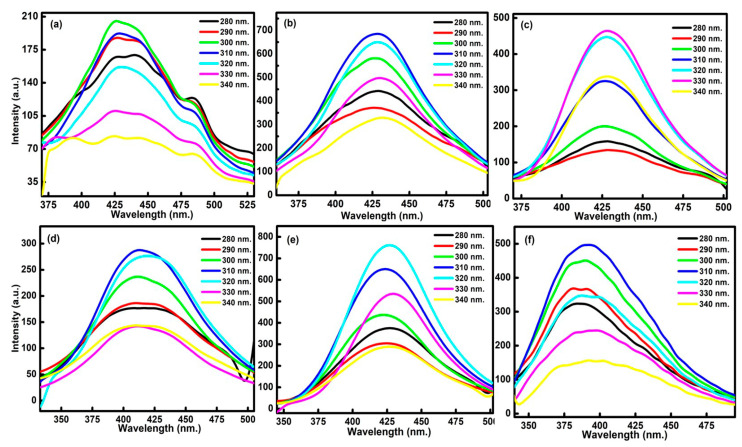
Excitation wavelength-dependent fluorescence spectrum of (**a**) P@C-dots, (**b**) C@C-dots, (**c**) B@C-dots, (**d**) P@ACs, (**e**) C@ACs and (**f**) B@ACs ranging from 280 nm to 340 nm.

Further, the emission behaviour of formed particles was analysed as a function of excitation wavelength between 280 to 380 nm for all the six types of formed particles (Figure 5). From the data, it was observed that the obtained emission intensity of formed particles is higher at 310 nm (λ_exc_). Further change in the λ_exc_ values produces decreasing influence on the emission intensity. The respective changes in the values are further aroused due to the different nature of defective bound excitons and trap states over the surface of formed particles [27]. These changes further affect the nature of vacant zig zag sites and available imprecations on formed C-dots and their counterpart ACs. 

### 3.3. Solvatochromic Properties of Carbon Dots and Their Activated Counterparts

#### 3.3.1. UV-Visible Spectroscopic Changes in C-Dots and ACs in Presence of Different Solvent

**Figure 6:**
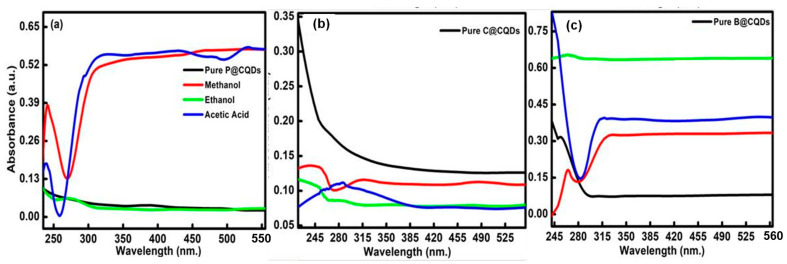
UV-visible spectra of (**a**) P@CQDs, (**b**) C@CQDs, (**c**) B@CQD in presence of polar protic solvents including methanol, ethanol and acetic acid.

**Figure 7:**
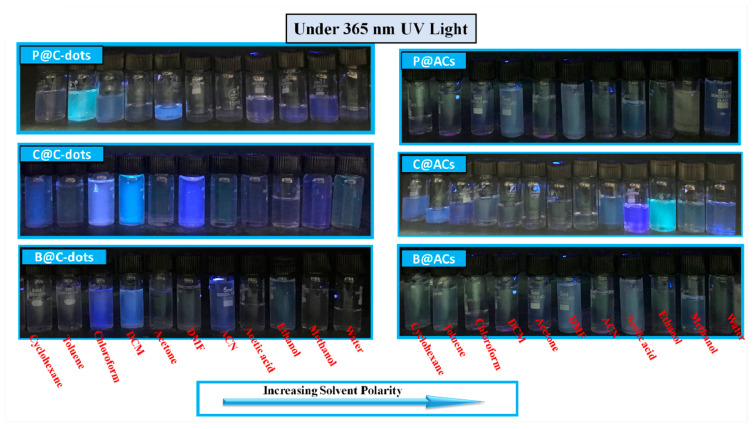
The digital images under UV light exposure for C-dots along with their counterpart ACs in presence of different solvents.

The existence of different types of functional groups over the exterior surface and the presence of conjugated systems in C-dots have the ability to induce solvatochromic behaviour in the presence of different solvents. Figure 6a–c displays the solvent-dependent absorbance spectra of C-dots prepared from poly-bags, plastic cups and bottles as precursor sources in polar protic solvents. The spectra for polar and non-polar solvents for C-dots are presented in Figure S2. However, the UV-visible spectra for ACs are shown in Figure S3. All the samples show the existence of shoulder peaks in the range from 220 to 500 nm (Figure 6). These peaks are aroused due to the transitions from minimum vibrational level in C-dots to different vibrational levels in excited states in π-conjugated structure of C-dots and their counter ACs. The digital images showing the variations of formed particles in different solvents are shown in Figure 7. 

The differences in the surface state of formed particles in different solvents are mainly responsible for the changes in the absorbance spectra of C-dots and ACs (Figure S3) [30,31]. The decreased absorbance value in different solvents as compared to water is explained due to the difference in the dispersibility rate of formed particles in those solvents. In addition, it has been observed that all the formed particles displayed the shifting in the peak in the presence of different solvents as compared to water, making them highly efficient materials in comparison to quantum dots and other types of luminogens. The peaks are quite sharp in the case of DMF for P@C-dots.

However, the nature of the peaks remains the same in the counterpart ACs. The blue shift in peaks on increasing the solvent polarity is further associated with the solvation effect generated in the presence of different solvents [32]. Additionally, the solvent molecules have the tendency to reorient around the surface of the excited state of C-dots and their counterpart ACs, which further have the tendency to reduce the energy gap between the ground and excited states of formed particles. However, the probability of hydrogen bonding among the solvents and the exterior surface of C-dots and their counterpart is significantly less in this case. Apart from the considerable difference in absorbance spectra, the three types of formed C-dots and their counterpart ACs have also shown distinct coloration in solution under the exposure of UV light (Figure S3). Additionally, the red shifting of absorbance peak in the case of C@C-dots in the presence of toluene has been observed in comparison to water as the dispersion medium. This behavioural change has been associated with the decrement in the energy gap in formed C-dots. The variation in the dipole moment with the solvent polarity also induced the changes in the surface electronic structure of formed C-dots and reduced the energy gap in formed particles and caused red shift in the absorbance peaks. The broadening of the peak in the presence of some solvents is mainly associated with the solvation effect in C-dots and ACs.

#### 3.3.2. Fluorescence Emission Changes in C-Dots and ACs in Presence of Different Solvent

The solvatochromic behavior of formed C-dots and their counterpart ACs was further studied by measuring their fluorescence emission spectra in a diverse range of solvents with varied polarity range (Figure S4). For the analysis, the formed particles of C-dots and their counterpart ACs were dissolved in 11 different solvents of different polarity. The dispersed particles showed a high range of solubility in all of the chosen solvents, which included polar protic (water, methanol, ethanol, acetic acid), polar aprotic (acetone, acetonitrile, dimethylformamide) and non-polar solvents (cyclohexane, dichloromethane, chloroform, toluene) [33]. The high range of solubility of formed particles in chosen solvents is a direct indication of the presence of hydrophilic and hydrophobic types of functional groups on the exterior surface of C-dots and their counterpart ACs. The emission profile in each case is recorded via exciting the sample at 310 nm (Figure S4). The spectra displayed the significant shifting in peak position via changing the solvent polarity in the dispersion medium. This behavioral aspect is further associated with the interaction of chromophores of formed C-dots and ACs with the solvent molecules. These interactions are mainly aroused due to the different extent of hydrogen bonding and dipole-based interaction of formed C-dots with solvent molecules [34]. Moreover, the variations in the electron density or the formation of new energy states in different types of solvents is also responsible for the solvent-dependent changes in the emission profile of C-dots and their counterpart ACs. Additionally, the changes in the energy gap also contribute towards the solvatochromic changes in the emission profile of formed C-dots and ACs.

For instance, in the case of P@C-dots, the emission peak for polar protic solvent was positioned at 425, 410, 405, 425 nm for water, methanol, ethanol and acetic acid, respectively. However, its counterpart P@ACs show the existence of emission peaks at 360, 345, 341, and 389 for water, methanol, ethanol and acetic acid as polar protic solvent medium, respectively (Figure S4a). The blue shifting of emission peaks is mainly associated with the change in the solvent polarity, which further affects the surface electronic states in P@ACs as compared to P@C-dots (Figure S4d). The corresponding enhancement in the energy gap is also responsible for the blue shifting in the fluorescence emission wavelength in P@C-dots and its counterpart ACs. However, it has been observed that there is a red shift in the emission peak of around 5, 7 and 10 nm for P@ACs in comparison to P@C-dots in the cases of acetone, ACN and DMF, respectively, as solvent medium. This variation mainly arose due to the competition of hydrogen bonding and dipole-based interaction between the solvent and formed C-dots and their counter ACs. In the case of non-polar solvent, the emission peaks were observed at 372, 371, 385, 350 nm for P@C-dots in cyclohexane, DCM, chloroform and toluene, respectively (Figure S4a,d). However, in the case of P@ACs, the respective peaks in these four non-polar solvents were observed at 359, 370, 409 and 342 nm, respectively. The peaks were observed at 387, 390, 401, 351 and 414 nm for C@C-dots in water, methanol, ethanol and acetic acid, respectively (Figure S4b). On the other hand, the peaks for C-@ACs were observed at 420, 412, 411 and 379 and 437 for water, methanol, ethanol and acetic acid, respectively (Figure S4e). 

The corresponding red shift of 33, 22, 10, 28 and 23 nm was mainly associated with the decrement in the energy gap of two different solvents. The positive increment in the FWHM value of emission peaks is mainly due to the solvation effect in a particular solvent. For aprotic solvents, the red shift of around 43 and 36 has been observed in the case of acetone and DMF as solvent for activated C-dots prepared from waste plastic cups as compared to C@C-dots. However, the blue shift of 28 nm has been observed in the case of ACN for C@ACs as compared to C@C-dots. This basic change is related to the variation in the refractive index (n_D_) of acetone, DMF and ACN as solvents. 

**Figure 8:**
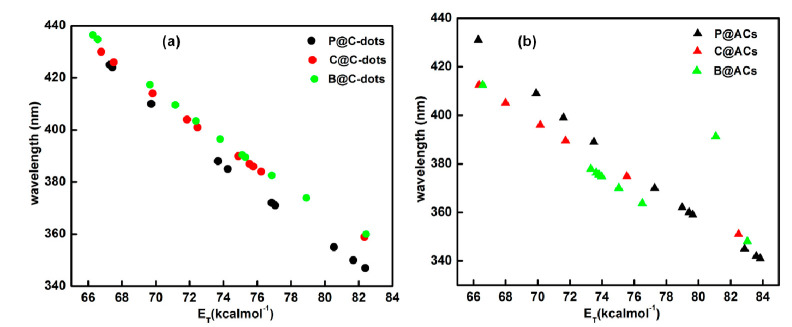
Variation of wavelength and E_T_ parameters for C-dots (**a**) and their counterpart ACs (**b**) derived from polybags, cups and bottles.

The lesser value of n_D_ in the case of ACN affected the stabilization of ground and excited states, whereas, in the case of acetone and DMF, these transition states are easily stabilized via the quick motion of electrons in the solvent molecules. Thus, the redistribution of electrons in transition states in acetone and DMF as solvent medium produced significant red shifting in the emission peaks and further reduced the energy difference between the excited and ground state. Additionally, the small range of red shifts in emission peaks of around ~10, 12, 3 and 49 nm was observed for C@ACs in cyclohexane, DCM, chloroform and toluene as solvent medium in comparison to C@C-dots (Figure S4b,e). Thus, the data support the better redistribution of electrons in the case of ACs in comparison to formed C-dots. For B@C-dots, the emission peak was observed at 429, 400, 433 and 343 nm for water, methanol, ethanol and acetic acid as solvent medium, respectively (Figure S4c). However, the counterpart AC showed the blue shift of around 41 nm in the case of water as solvent medium. The blue shifts of 9 nm and 4 nm were observed in the case of methanol and ethanol, respectively, for B@ACs (Figure S4f). This small blue shifting of emission peak is mainly associated with the change in the solvent polarity and its influence on the surface electronic states in B@ACs as compared to B@C-dots. However, the small red shifting of peaks was observed for B@ACs in the case of polar aprotic and non-polar solvents. Moreover, these changes in the emission peaks in various chosen solvents displayed a higher range of accessible interaction of prepared particles with solvent molecules and enhanced the scope of prepared particles in different ranges of solvents varying from polar to polar aprotic to non-polar categories. To explore the solvatochromic properties of the formed C-dots and their ACs in different solvents, we further calculated the molar electronic transition energy values (E_T_) using Equation (1) (Figure 8) [35]:

(1) $$E_{T}(KCalmol^{-1})=\frac{hcN_{A}}{\lambda_{\max}{\left(nm\right)}^{}}=\frac{28,591}{\lambda_{\max}(nm)}$$

The linear association of emission maximum with E_T_ values in different solvents clearly supports the solvatochromic properties of the formed C-dots and their ACs and enhances its scope in sensing of different analytes in a range of solvents.

## 4. Conclusions

To summarize, the detailed solvatochromic properties of three different novel types of C-dots and their counterpart activated form have been investigated and compared. The respective variations in the spectra clearly support the differences in the surface state of formed particles. These differences in the transition states further contribute to the variations in the interactions between surface structures of formed particles with the chosen solvent molecules. By detailed investigation of emission properties, we can conclude that the changes in the ground and excited state energy are mainly affected by the solvent system, which further influenced the red and blue shift in the emission wavelength. This detailed study experimentally establishes the important solvatochromic behaviors for C-dots and their counterpart ACs in polar protic, aprotic and non-polar solvents. The results broaden the application scope of developed C-dots and their ACs and can inform the customized design of new nano-probes with intelligent responsive behaviour in different solvents.

## Data Availability

The data will made available on request.

## Supplementary Materials

The following supporting information can be downloaded at: https://www.mdpi.com/article/10.3390/nano13081398/s1. Figure S1. XRD profile of (a) C-dots and (b) ACs derived from single use plastic bottle, cups and polybags; Figure S2. UV-visible spectra of (a,b) P@CQDs, (c,d) C@CQDs and (e,f) B@CQDs in presence of protic and non-polar solvents; Figure S3. UV-visible spectra of (a–c) P@ACs, (d–f) C@ACs and (g–i) B@ACs in presence of protic and non-polar solvents; Figure S4. Fluorescence emission spectrum of (a) P@C-dots, (b) C@C-dots, (c) B@C-dots, (d) P@ACs, (e) C@ACs and (f) B@ACs in presence of different solvents; Table S1. Illustration of different parameters obtained from FTIR spectrum for synthesized CQDs and ACs derived from waste plastic; Table S2. Representation of different parameters calculated from XRD analysis for developed C-dots and ACs from Plastic waste; Table S3. The corresponding positioning, area under the curve and the FWHM for four different kinds of CQDs.
