# Supplementary material for: Solvatochromism as a Novel Tool to Enumerate the Optical and Luminescence Properties of Plastic Waste Derived Carbon Nanodots and Their Activated Counterparts
Source: Nanomaterials (Basel). 2023 Apr 18;13(8):1398. doi: 10.3390/nano13081398 (PMC10143849; doi:10.3390/nano13081398)
Supplement: Supplementary file 1 [file nanomaterials-13-01398-s001.zip › nanomaterials-2305692-supplementary.doc]

Supplementary file

Solvatochromism as a Novel Tool to Enumerate the Optical and Luminescence Properties of Plastic Waste Derived Carbon
Nanodots and Their Activated Counterparts

Savita Chaudhary ^1,^*, Manisha Kumari ^1^, Pooja Chauhan ^1^, Ganga Ram Chaudhary ^1^, Ahmad Umar ^2,3,4,^*^,†^,
Sheikh Akbar ^4^ and Sotirios Baskoutas ^5^

^1^	Department of Chemistry and Centre of Advanced Studies in Chemistry, Panjab University,
Chandigarh 160014, India; mannatpatyal@gmail.com (M.K.); pujachauhan.05@gmail.com (P.C.); grc22@pu.ac.in (G.R.C.)

^2^	Centre for Scientific and Engineering Research, Najran University, Najran 11001, Saudi Arabia

^3^	Department of Chemistry, Faculty of Science and Arts, and Promising Centre for Sensors and Electronic Devices (PCSED), Najran University, Najran 11001, Saudi Arabia

^4^	Department of Materials Science and Engineering, The Ohio State University, Columbus, OH 43210, USA; akbar.1@osu.edu (S.A.)

^5^	Department of Materials Science, University of Patras, 26500 Patras, Greece; bask@upatras.gr (S.B.)

*****	Correspondence: schaudhary@pu.ac.in (S.C.); ahmadumar786@gmail.com (A.U.)

†	Adjunct Professor at the Department of Materials Science and Engineering, The Ohio State University,
Columbus, OH 43210, USA.

**Table S1.** Illustration of different parameters obtained from FTIR spectrum for synthesized CQDs and ACs derived from waste plastic.

| **S. No.** | **Absorption peak (cm^-1^)** | **Bond Assignment** | **Appearance** |
| --- | --- | --- | --- |
| **1.** | 3400-3500 cm^-1^ | -OH Stretching | Strong |
| **2.** | 1628 cm^-1^ | -C=C Stretching | Strong |
| **3.** | 1414 cm^-1^ | –C-OH stretching | Medium |
| **4.** | 700 cm^-1^ | C=C bending | Medium |
| **5.** | 1600 cm^-1^ | C-C, C=O and C=C stretching | Strong |

**Figure S1.** XRD profile of (a) C-dots and (b)ACs derived from single use plastic bottle, cups and polybags.

**Figure S2.** UV-visible spectra of (a-b) P@CQDs, (c-d) C@CQDs and (e-f) B@CQDs in presence of protic and non-polar solvents. (r) The digital images under UV light exposure for C-dots along with their counter ACs in presence of different solvents.

**Figure S3.** UV-visible spectra of (a-c) P@ACs, (d-f) C@ACs and (g-i) B@ACs in presence of protic and non-polar solvents. (r) The digital images under UV light exposure for C-dots along with their counter ACs in presence of different solvents.

**Figure S4.** Fluorescence emission spectrum of (a) P@C-dots, (b) C@C-dots, (c) B@C-dots, (d) P@ACs, (e) C@ACs and (f) B@ACs in presence of different solvents.

**Table S2.** Representation of different parameters calculated from XRD analysis for developed C-dots and ACs from Plastic waste.

| **System** | **Peak at 2θ** | **Miller Indices** | **Size (nm)** |
| --- | --- | --- | --- |
| **P@C-dots** | 21.4, 23.84, 29.46, 31.2 and 36.25 | (100), (101), (102), (201) and (105) | 20 |
| **C@ C-dots** | 12.2, 24.65, 29.66, 30.66 and 35.67 | (100), (002), (201), (107) and (200) | 31 |
| **B@ C-dots** | 11.82, 20.64, 29.46 and 29.46 | (100), (101), (105) and (111) | 28 |
| **P@ACs** | 21.25, 23.84, 29.66, 36.28, 39.48, 43.27 and 47.7 | (002), (105), (006), (107), (207, (206) and (304) | 24 |
| **C@ACs** | 24.4, 29.46, 31.47, 32.86 and 34.06 | (002), (201), (111), (101) and (206) | 34 |
| **B@ACs** | 21.64 | (100) | 31 |

**Table S3.** The corresponding positioning, area under the curve and the FWHM for four different kinds of CQDs.

| **Sample** | **Peak one**  **Defect-bound excitons (1_DBE_).** | | | **Peak 2**  **Defect-charged excitons (2_DCE_)** | | | **Peak 3**  **Electron–hole recombination effect (3_EHR_)** | | |
| --- | --- | --- | --- | --- | --- | --- | --- | --- | --- |
|  | **Peak position (nm)** | **Area** | **FWHM** | **Peak position (nm)** | **Area** | **FWHM** | **Peak position**  **(nm)** | **Area** | **FWHM** |
| **P@C-dots** | 405.8 | 5.7 x 10^3^ | 66.9 | 429.5 | 4.3 x 10^3^ | 40.6 | 469.3 | 5.4 x 10^3^ | 56.7 |
| **C@C-dots** | 406.7 | 17.7 x 10^3^ | 78.9 | 426.1 | 19.3 x 10^3^ | 48.4 | 450.8 | 8.9 x 10^2^ | 89.4 |
| **B@C-dots** | 410.3 | 8.7 x 10^2^ | 21.1 | 430.1 | 25.9 x 10^3^ | 48.4 | 480.6 | 2.9 x 10^3^ | 36.2 |
| **P@ACs** | 396.3 | 4.6 x 10^2^ | 23.3 | 417.6 | 27.2 x 10^3^ | 76.6 | 499.7 | 3.2 x 10^3^ | 64.7 |
| **C@ACs** | 397.1 | 2.5 x 10^3^ | 31.7 | 424.6 | 36.7 x 10^3^ | 49.9 | 456.8 | 19.3 x 10^3^ | 74.7 |
| **B@ACs** | 375.9 | 3.75 x 10^2^ | 45.7 | 409.5 | 21.9 x 10^3^ | 59.6 | 451.1 | 8.6 x 10^3^ | 87.9 |
